# Impact of seasonality and malaria control interventions on *Anopheles* density and species composition from three areas of Uganda with differing malaria endemicity

**DOI:** 10.1186/s12936-021-03675-5

**Published:** 2021-03-07

**Authors:** Henry Ddumba Mawejje, Maxwell Kilama, Simon P. Kigozi, Alex K. Musiime, Moses Kamya, Jo Lines, Steven W. Lindsay, David Smith, Grant Dorsey, Martin J. Donnelly, Sarah G. Staedke

**Affiliations:** 1 Infectious Diseases Research Collaboration, Kampala, Uganda; 2 Department of Medicine, Makerere University College of Health Sciences, Kampala, Uganda; 3 London School of Hygiene and Tropical Medicine, London, UK; 4 Department of Vector Biology, Liverpool School of Tropical Medicine, Pembroke Place Liverpool, UK; 5 Department of Medicine, University of California, San Francisco, USA; 6 Department of Biosciences, Durham University, Durham, UK; 7 Department of Health Metrics Sciences, University of Washington, Seattle, WA USA

**Keywords:** Seasonality, Malaria control, Interventions, *Anopheles* density, Species composition

## Abstract

**Background:**

Long-lasting insecticidal nets (LLINs) and indoor residual spraying (IRS) are the malaria control interventions primarily responsible for reductions in transmission intensity across sub-Saharan Africa. These interventions, however, may have differential impact on *Anopheles* species composition and density. This study examined the changing pattern of *Anopheles* species in three areas of Uganda with markedly different transmission intensities and different levels of vector control.

**Methods:**

From October 2011 to June 2016 mosquitoes were collected monthly using CDC light traps from 100 randomly selected households in three areas: Walukuba (low transmission), Kihihi (moderate transmission) and Nagongera (high transmission). LLINs were distributed in November 2013 in Walukuba and Nagongera and in June 2014 in Kihihi. IRS was implemented only in Nagongera, with three rounds of bendiocarb delivered between December 2014 and June 2015. Mosquito species were identified morphologically and by PCR (Polymerase Chain Reaction).

**Results:**

In Walukuba, LLIN distribution was associated with a decline in *Anopheles funestus* vector density (0.07 *vs* 0.02 mosquitoes per house per night, density ratio [DR] 0.34, 95% CI: 0.18–0.65, *p* = 0.001), but not *Anopheles gambiae *sensu stricto (*s.s.)* nor *Anopheles arabiensis*. In Kihihi, over 98% of mosquitoes were *An. gambiae s.s*. and LLIN distribution was associated with a decline in *An. gambiae s.s.* vector density (4.00 *vs* 2.46, DR 0.68, 95% CI: 0.49–0.94, *p* = 0.02). In Nagongera, the combination of LLINs and multiple rounds of IRS was associated with almost complete elimination of *An. gambiae s.s.* (28.0 *vs* 0.17, DR 0.004, 95% CI: 0.002–0.009, *p* < 0.001), and *An. funestus *sensu lato (*s.l*.) (3.90 *vs* 0.006, DR 0.001, 95% CI: 0.0005–0.004, *p* < 0.001), with a less pronounced decline in *An. arabiensis* (9.18 *vs* 2.00, DR 0.15 95% CI: 0.07–0.33, *p* < 0.001)*.*

**Conclusions:**

LLIN distribution was associated with reductions in *An. funestus s.l.* in the lowest transmission site and *An. gambiae s.s.* in the moderate transmission site. In the highest transmission site, a combination of LLINs and multiple rounds of IRS was associated with the near collapse of *An. gambiae s.s.* and *An. funestus s.l.* Following IRS, *An. arabiensis*, a behaviourally resilient vector, became the predominant species, which may have implications for malaria vector control activities. Development of interventions targeted at outdoor biting remains a priority.

## Background

Over the past two decades, improved funding and intensive malaria control efforts have increased coverage of vector control interventions worldwide, chiefly long-lasting insecticidal nets (LLINs) and indoor residual spraying (IRS) [1–3]. Within this period, a significant decline in the burden of malaria has been reported across sub-Saharan Africa, with most of this reduction attributed to LLINs (68%), and to a lesser extent, use of IRS (13%) [1]. Global progress toward reducing the incidence of malaria and related deaths, however, has stalled recently [3]. In response, the World Health Organization (WHO) has called for a locally-tailored approach to malaria control rather than a ‘one size fits all’ policy [3].

In Uganda, focused efforts to ensure universal coverage of LLINs through mass distribution campaigns have increased household ownership of LLINs, from 47% in 2009 to over 80% in 2015 and 2019 [4–6]. IRS has also been implemented, beginning with 10 districts from 2007 to 2014, and moving to 14 new districts in 2014 [5, 7–9]. Concomitantly, malaria prevalence has declined in children under five years old, from 40% in 2009, to 19% in 2015 [5], and, further, to 9% in 2019 [6]. In Uganda [10], Kenya [11] and elsewhere [12], sustained vector control has not only resulted in reductions in transmission intensity, but also changes in *Anopheles* species composition, their behaviour [13, 14], and density [15].

*Anopheles gambiae *sensu lato (*s.l.)* and *Anopheles funestus s.l.* are the primary malaria vector groups in Uganda [4, 16], and elsewhere in East Africa [17, 18]. Both groups are species complexes, comprising of genetically distinct but morphologically indistinguishable sibling species [19–23]. In the *An. gambiae* complex, *An. gambiae *sensu stricto (*s.s*.) and *An. arabiensis* differ in several aspects, including breeding environment, host preference, biting behaviours, malaria infection rates, and insecticide resistance patterns [14, 17, 24]. *Anopheles gambiae s.s.* prefer to feed on humans and rest indoors [17]. In contrast, *An. arabiensis* is less anthropophilic [25, 26]; feeding preferences vary with host availability across the species range [27, 28], with exophilic tendencies [29, 30]. In some mosquito populations, *An. gambiae s.s.* has higher *Plasmodium falciparum* infection rates [31], and higher levels of pyrethroid resistance [32], than *An. arabiensis.* Hybrids between *An. gambiae s.s.* and *An. arabiensis* have also been identified [33, 34], with evidence of gene flow between the two species [34]. The implication of hybrids for malaria control is still poorly understood, although in some populations adaptive introgression of insecticide resistance genes coincident with LLIN distribution has been observed [35]. In contrast, *An. funestus s.l.* breeds year-round in stable environments, such as marshland [20, 36], and may engage in early-morning biting [37]. *Anopheles funestus s.l.* remains an important vector in dry seasons as a result of its breeding habits [38, 39].

With the expansion of vector control, changes in *Anopheles* species composition and mosquito density have been observed in Uganda [10, 15], and elsewhere in sub-Saharan Africa [26, 40, 41]. Changes in malaria vector species composition in response to vector control interventions are not a new phenomenon and have been described previously [42]. Recent studies have demonstrated an increase in the relative abundance of *An. arabiensis,* when compared to sympatric *An. gambiae s.s.* following deployment of LLINs and/or IRS [10, 11, 14]. Similarly, the apparent replacement of highly anthropophilic *An. funestus s.s.* by less anthropophilic (zoophilic) and more exophilic *Anopheles rivulorum* in response to IRS in neighbouring Tanzania, was observed in the *An. funestus s.l.* complex in the 1960s [42]. Due to their more zoophilic and exophilic behaviour, vector control interventions have been less effective in controlling certain malaria vector species, such as *An. arabiensis* [41, 43], and *An. rivolurum* [42]. To further explore the species-specific impact of vector control interventions, the impact of LLINs and IRS on sympatric *An. gambiae s.s*., *An. arabiensis* and *An. funestus s.l.* was examined on mosquito density in areas with differing malaria endemicity in Uganda.

## Methods

### Study sites

**Fig. 1:**
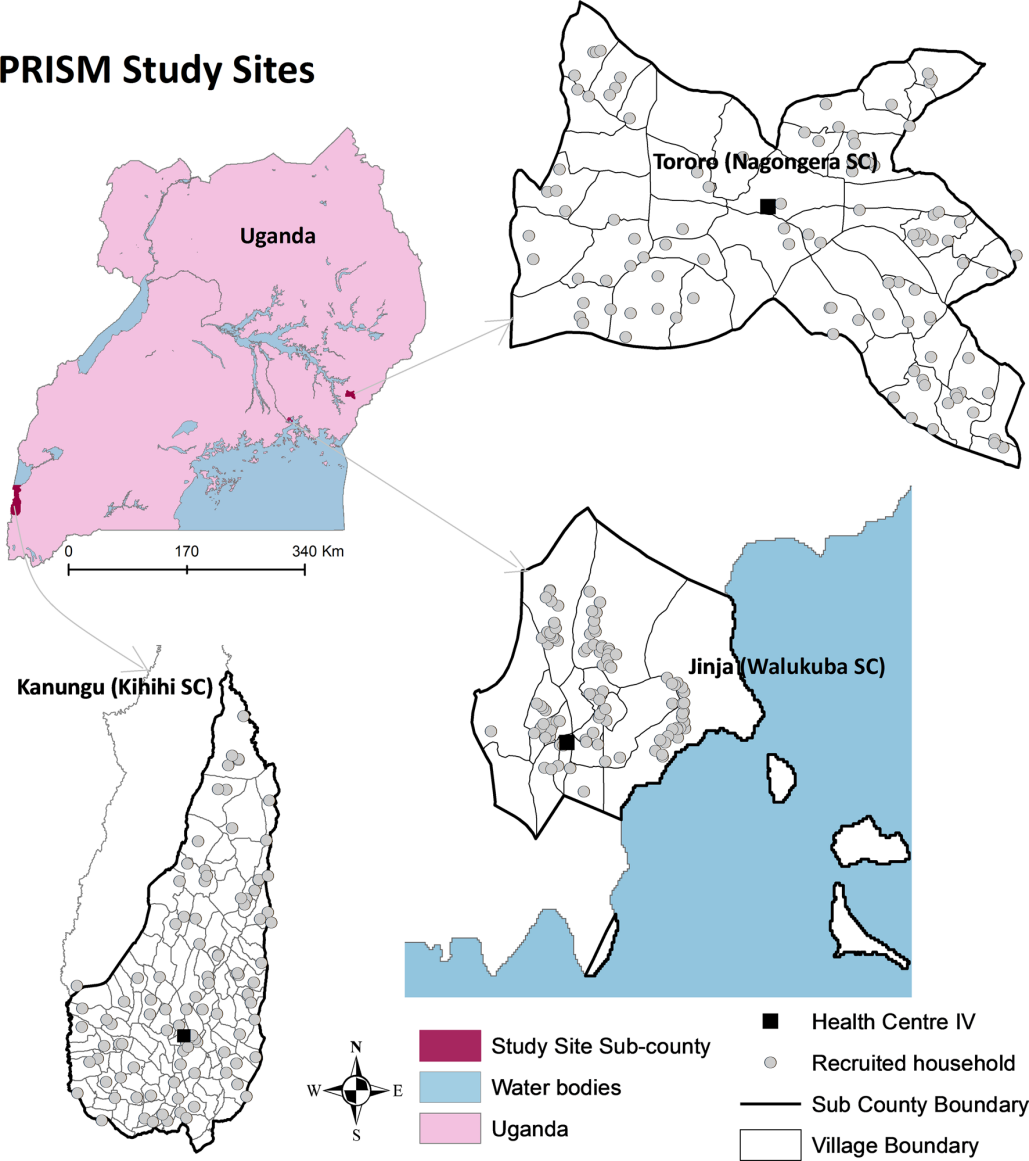
Map of Uganda showing study site location. Grey dots show location of households sampled for CDC light trap collections in the PRISM cohort (Programme for Resistance, Immunology, Surveillance and Modelling of Malaria). Image from Kigozi et al. [45]

This study was conducted from October 2011 to June 2016 in three sites with differing malaria endemicity, within Walukuba, Kihihi and Nagongera sub-counties (Fig. 1), as part of the PRISM1 (Programme for Resistance, Immunology, Surveillance and Modelling of Malaria) project [10, 44, 45] [46]. Walukuba sub-county (00°26′33.2″N, 33°13′32.3″E), located on the fringes of Lake Victoria in Jinja District, eastern Uganda is a peri-urban area at an elevation of 1,215 m with low malaria transmission [baseline annual human biting rate of 537 and *P. falciparum* entomological inoculation rate (EIR) of 3.2 infective bites per person per year] [46, 47]. *Anopheles arabiensis* has been the predominant malaria vector species in this area [46, 48]. Kihihi sub-county (00°45′03.1″S, 29°42′03.6″E), located in Kanungu District, southwestern Uganda, is a rural and hilly area 1,310 m above sea level, with moderate malaria transmission (baseline annual human biting rate of 1,337 and *P. falciparum* EIR of 14.2 infective bites per person per year) [46]. *Anopheles gambiae s.s.* has been the main malaria vector species in Kihihi [46, 48]. Nagongera sub-county (00°46′10.6″ N, 34°01′34.1″ E), located in Tororo District, eastern Uganda, is a rural area bordering Kenya with an elevation of 1,185 m with high malaria transmission (baseline annual human biting rate of 16,606 reported in 2014 and *P. falciparum* EIR of 310 infective bites per person per year) [46]. *Anopheles gambiae s.s*. has been described as the main malaria vector in Tororo [48], however, in 2014 increasing proportions of *An. arabiensis* were documented [46]. Seasonality in Uganda is characterized by alternating rainy and dry seasons and a bimodal rainfall pattern. The longer rainy season occurs between July and November and the shorter rainy season between February and May [33].

During 2011–2016, the primary malaria control interventions deployed in Uganda included artemisinin-based combination therapy for treatment of uncomplicated malaria, distribution of LLINs through mass campaigns, and IRS in select districts [5]. LLINs were delivered to Walukuba and Nagongera in November 2013, and to Kihihi in June 2014. In Nagongera, three rounds of IRS with a carbamate insecticide (bendiocarb) were implemented between December 2014 and June 2015 (1st: December 2014 to Feb 2015, 2nd: June-July 2015, and 3^rd^: November–December 2015).

### Household selection

**Fig. 2:**
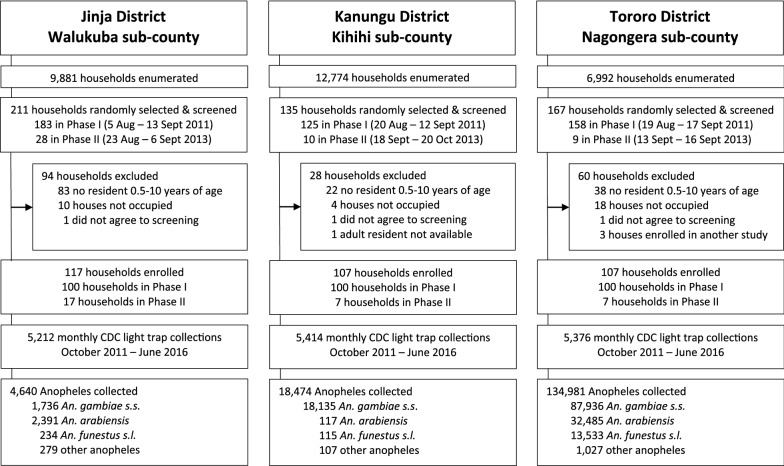
Study profile of Walukuba, Kihihi and Nagongera sub counties

During the initial enrollment period in 2011, 100 households per site were randomly selected from a list of enumerated of households, as previously described [44]. In 2013, additional households were enrolled to replace households that had dropped out of the study to increase the number of enrolled households back to 100 per site (Fig. 2).

### Mosquito collection

Mosquitoes were collected monthly from cohort study households using miniature CDC light traps (Model 512; John W. Hock Company, Gainesville, FL, USA) set at 19:00 h and collected the following morning at 07:00 h. One trap was set per household each month from October 2011 to June 2016. Light traps were positioned indoors, 1 m above the ground at the foot end of the bed, next to a study participant, sleeping under a LLIN [46]. Data were excluded from analysis if the target occupant did not sleep in the selected room or if the light trap was faulty.

### Mosquito species identification

All anophelines collected were scored morphologically under dissecting microscopes at the study sites using taxonomic keys [21, 49]. A subset of 30–50 mosquitoes was randomly selected per month per site for the entire study period for purposes of identifying members of the *An. gambiae* species complex using PCR [50]. The *An. funestus* species complex was not processed beyond morphological identification due to resource limitations (henceforth referred to as *An. funestus*). Results from the species identification were extrapolated to the total dataset to establish the species composition of all *Anopheles* collected at each site every month. Approximately, 10% of the *Anopheles* collected were non-malaria transmitting *Anopheles christyi*, classified as ‘other *Anopheles* species’ and were not processed further.

### Data management and analysis

Field entomologists recorded CDC light trap data on standardized forms. The data collection forms were double-entered into a Microsoft Access database and checked for discrepancies. Any subsequent inconsistencies were resolved using original data entry forms. Statistical analysis was done using Stata (version 14.2, Stata Corp, College Station, TX, USA).

The primary independent variables investigated were; seasonality (dry *versus* wet season) and the combined vector control interventions (pre-intervention *versus* post-intervention). The outcomes of interest were vector density and species composition. Seasonality, denoted by rainy and dry seasons was generated for each site independently. For each site, the same consecutive months were divided into 2 rainy seasons and 2 dry seasons over 1 calendar year. Months with rainfall above and below the median value for the entire observation period were classified as rainy or dry season, respectively, after including a 1-month lag period. Vector density was determined by the number of mosquitoes collected per household per month per site and stratified by seasonality and the period before intervention implementation *versus* the period after intervention implementation. Simple proportions were compared using a log-binomial regression model with generalized estimating equations to adjust for repeated measures from the same house.

Here, we expand on the PRISM1 results previously reported by Kilama et al*.* [46] from observations carried out over 12 months (October 2011 to September 2012), by describing species-specific changes in response to vector control interventions carried out over 57 months (October 2011 to June 2016). Musiime et al*.* also used PRISM1 data to examine the impact of vector control interventions on *Anopheles* mosquito composition in Nagongera only, as measured using indoor and outdoor human landing catches [10]. This study analyses mosquitoes collected indoors using CDC light traps using longitudinal sampling in the three study sites. The PRISM1 dataset can be accessed at https://clinepidb.org/ce/app/record/dataset/DS_0ad509829e.

### Ethical approval and consent

In each study site, the head of household or adult representative was approached for consenting before household recruitment. A written informed consent was obtained as permission to conduct CDC light trap collections within the household. The study was approved by the Uganda National Council for Science and Technology (HS-119ES), Makerere University School of Medicine Research and Ethics Committee (2017-099), the University of California, San Francisco Committee on Human Research (17-22544) and London School of Hygiene and Tropical Medicine Ethics Comittee (14266-6).

## Results

### Total *Anopheles* mosquitoes collected

From October 2011 to June 2016, 16,002 light trap collections were performed monthly across the three study sites. Overall, 158,095 *Anopheles* mosquitoes were collected, including 4,640 (3%) from Walukuba, 18,474 (12%) from Kihihi, and 134,981 (85%) from Nagongera (Table 1, Fig. 2). The number of *Anopheles* mosquitoes collected per household per night (vector density) varied across the sites from 0.89 in Walukuba to 25.11 in Nagongera (Table 1). Overall, *An. arabiensis* (n = 2,391) was the predominant malaria vector species in Walukuba accounting for 52% of all collections. In Kihihi, nearly all *Anopheles* collected (98%) were *An. gambiae s.s.* (n = 18,135), while in Nagongera, 65% were *An. gambiae s.s.* (n = 87,936) (Table 1). Of the 1,413 ‘other’ *Anopheles* species collected in the sites, 1,385 (98%) were identified morphologically as *An. christyi,* which is classified as a non-malaria vector [51]. There is historical evidence that *An. christyi* has the ability to transmit malaria parasites [52], however, subsequent reports argue that this ability was either lost or suppressed independently [51] and is thus now considered to be a non-malaria vector. As expected, more *Anopheles* mosquitoes were collected during rainy seasons, compared to the dry seasons (Table 2).

**Table 1:** Characteristics of sites and collections

	Walukuba	Kihihi	Nagongera
District	Jinja	Kanungu	Tororo
Entomological Inoculation Rate (EIR)^a^	3.2	14.2	310.0
Transmission intensity at baseline	Low	Medium	High
Households sampled (N)	5212	5414	5376
Total Anopheles collected (n)	4.640	18,474	134,981
Vector density	0.89	3.41	25.11
Mosquito collections			
*An. gambiae* s.s. (n, %)	1736 (37%)	18,135 (98%)	87,936 (65%)
*An. arabiensis* (n, %)	2391 (52%)	117 (0.6%)	32,485 (24.2%)
*An. funestus* s.l*.* (n, %)	234 (5%)	115 (0.6%)	13,533 (10%)
Other Anopheles (n, %)	279 (6%)	107 (0.6%)	1,027 (0.8%

^a^Infectious bites per person per year

**Table 2:** Stratified analysis of vector density (density ratio) by seasonality and intervention period

Study site	Variable	Categories	*An. gambiae s.s*			*An. arabiensis*			*An. funestus*		
			Vector density	DR (95% CI)	*p*-value	Vector density	DR (95% CI)	*p*-value	Vector density	DR (95% CI)	*p*-value
Walukuba^1^	Seasonality	Dry seasons^a^	0.16	Reference		0.22	Reference		0.02	Reference	
		Rainy seasons^b^	0.49	3.21 (2.15–4.79)	< 0.001	0.67	2.84 (1.87–4.32)	< 0.001	0.06	2.57 (1.36–4.88)	0.004
	Intervention Period	Before LLINs^c^	0.34	Reference		0.58	Reference		0.07	Reference	
		After LLINs^d^	0.33	1.29 (0.86–1.93)	0.21	0.35	0.85 (0.56–1.30)	0.45	0.02	0.34 (0.18–0.65)	0.001
Kihiihi^2^	Seasonality	Dry seasons^e^	0.78	Reference		Insufficient number of *An. arabiensis* collected			Insufficient number of *An. funestus s.l* collected		
		Rainy seasons^f^	4.50	5.56 (3.90–7.92)	< 0.001						
	Intervention Period	Before LLINs^g^	4.00	Reference		Insufficient number of *An. arabiensis* collected			Insufficient number of *An. funestus s.l* collected		
		After LLINs^h^	2.46	0.68 (0.49–0.94)	0.02						
Nagongera^3^	Seasonality	Dry seasons^i^	2.92	Reference		1.54	Reference		2.24	Reference	
		Rainy seasons^j^	25.4	12.2 (7.05–21.3)	< 0.001	9.06	7.75 (4.21–14.3)	< 0.001	2.70	1.61 (0.97–2.66)	0.06
	Intervention Period	Before LLINs^k^	28.0	Reference		9.18	Reference		3.90	Reference	
		After LLINs^l^	11.2	0.40 (0.21–0.73)	< 0.003	3.65	0.36 (0.18–0.72)	0.004	2.56	0.61 (0.36–1.04)	0.07
		After 1st Round of IRS^m^	2.71	0.05 (0.02–0.16)	< 0.001	5.44	0.33 (0.10–1.09)	0.07	0.10	0.02 (0.008–0.06)	< 0.001
		After 2nd Round of IRS^n^	0.17	0.004 (0.002–0.009)	< 0.001	2.00	0.15 (0.07–0.33)	< 0.001	0.006	0.001 (0.0005–0.004)	< 0.001

^1^Low malaria transmission

^2^Moderate malaria transmission

^3^High malaria transmission

^a^ February–April, July–September

^b^ May–June, October–January

^c^ Oct 2011–Nov 2013

^d^ Dec 2013–June 2016

^e^ January–February, July–August

^f^ March–June, September–December

^g^ Oct 2011–June 2014

^h^ July 2014–June 2016

^i^ January-March, August–September

^j^ April-July, October–December

^k^ Oct 2011–Nov 2013

^l^ Dec 2013–Feb 2015

^m^ March 2015–June 2015

^n^ July 2015–June 2016

### Trends in *Anopheles* mosquitoes in Walukuba

**Fig. 3:**
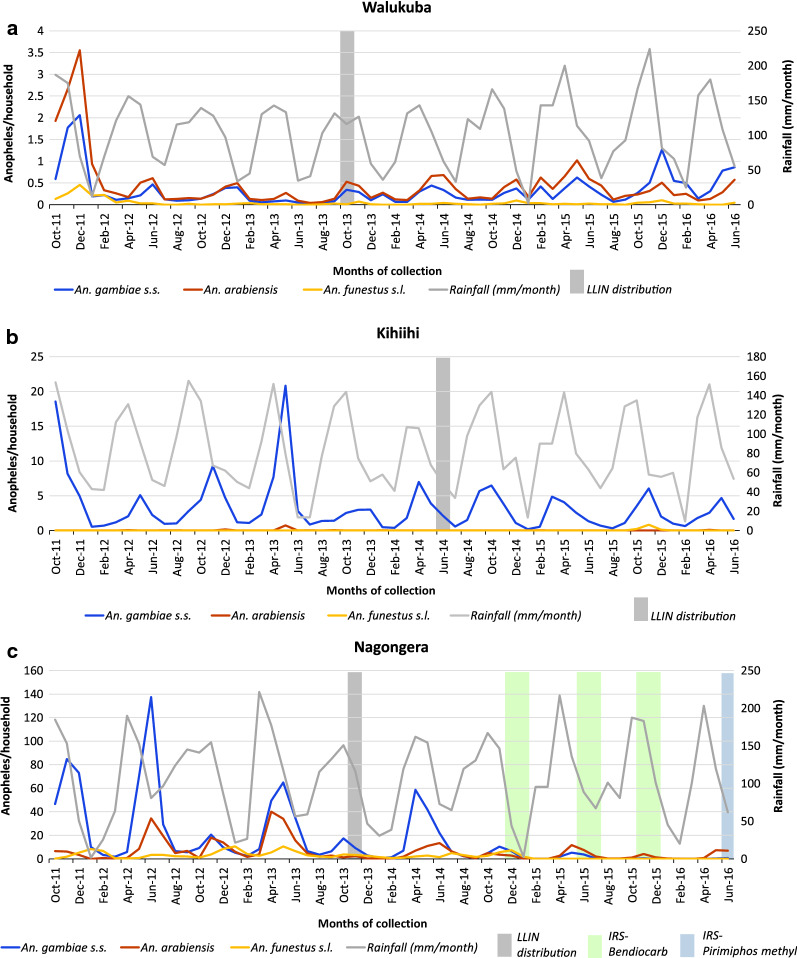
Absolute numbers of *An. gambiae s.s.* (blue line), *An. arabiensis* (red line) and *An. funestus s.l.* (yellow line), collected per month in the three study sites. The grey line shows the rainfall pattern, the grey bar depicts LLIN distribution and the green bars depict IRS deployment

**Fig. 4:**
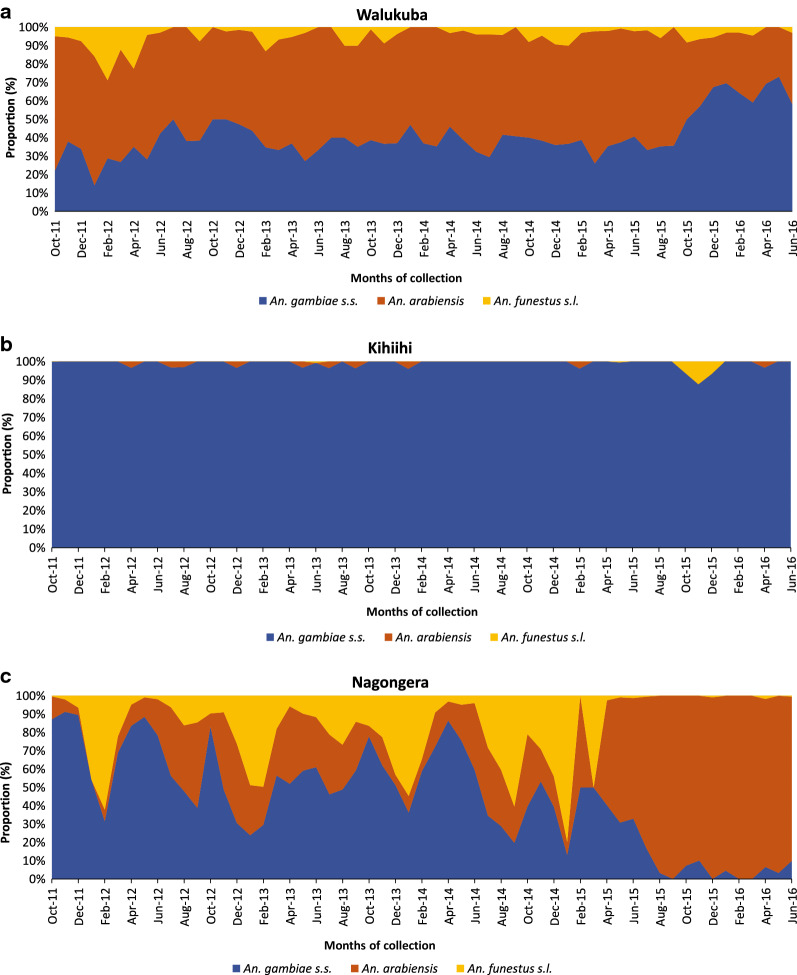
Relative numbers / proportion of *An. gambiae s.s* (blue), *An. arabiensis* (red) and *An. funestus s.l.* (yellow), collected per month in the three study sites

In Walukuba, the rainy season was associated with approximately a three-fold increase in vector density for all three main vectors, including *An. gambiae s.s.* (density ratio [DR] 3.21, 95% confidence interval [CI]: 2.15–4.79), *An. arabiensis* (DR 2.84, 95% CI: 1.87–4.32) and *An. funestus* (DR 2.57, 95% CI: 1.36–4.88; Table 2). Following LLIN distribution, approximately a threefold decline in *An. funestus* vector density (DR 0.34, 95% CI: 0.18–0.65; Table 2) was observed in Walukuba. The density of *An. gambiae s.s.* or *An. arabiensis* following distribution of LLINs was similar to levels before deployment (Table 2). This corresponded with the pattern of distribution observed in the graphical plots examining the absolute numbers of *Anopheles* collected in Walukuba (Fig. 3a) and the relative proportions (Fig. 4a) of mosquito species.

### Trends in *Anopheles* mosquitoes in Kihihi

In Kihihi, the rainy season was associated with over a five-fold increase in *An. gambiae s.s.* density (DR 5.56, 95% CI: 3.90–7.92) compared to the dry season. Insufficient numbers of both *An. arabiensis* and *An. funestus* were collected however, precluding further analysis. LLIN distribution in this area was associated with a decrease in *A. gambiae s.s.* vector density (DR 0.68, 95% CI: 0.49–0.94). This observation is supported by the longitudinal patterns for absolute numbers of *Anopheles* mosquitoes collected per household (Fig. 3b). When focusing only on trends in relative proportions of *Anopheles* over time, however, this finding is not obvious (Fig. 4b).

### Trends in *Anopheles* mosquitoes in Nagongera

In Nagongera, there were substantially more *An. gambiae s.s.* (DR 12.2, 95% CI: 7.05–21.3) and *An. arabiensis* (DR 7.75, 95% CI 4.21–14.3) during the rainy season, but no significant difference was observed for *An. funestus* (DR 1.61, 95% CI: 0.97–2.66). LLINs were associated with a significant decrease in vector density for *An. gambiae s.s.* (DR 0.40, 95% CI: 0.21–0.73) and *An. arabiensis* (DR 0.36, 95% CI 0.18–0.72), but not *An. funestus* (DR 0.61, 95% CI: 0.36–1.04). In Nagongera, three rounds of IRS with bendiocarb were delivered following LLIN distribution. The first round of IRS was associated with a 20-fold decline in *An. gambiae s.s.* vector density compared to the pre-LLIN period *(*DR 0.05, 95% CI: 0.02–0.16), while the impact on *An. funestus* was close to elimination (DR 0.02, 95% CI: 0.008–0.06). There was no difference in *An. arabiensis* densities before and after the first round of IRS (DR 0.33, 95% CI: 0.10–1.09). The 2nd and 3rd rounds of IRS (combined) were associated with further declines in vector density for both *An. gambiae s.s.* (DR 0.004, 95% CI: 0.002–0.009), and *An. funestus* (DR 0.001, 95% CI: 0.0005–0.004), but a less pronounced decline was observed in *An. arabiensis* vector density (DR 0.15, 95% CI: 0.07–0.33). In contrast to Walukuba and Kihihi, substantial reductions in the absolute numbers of *An. gambiae s.s.* and *An. funestus s.l.* were observed following the addition of IRS to LLINs (Fig. 3c). The absolute number of *An. arabiensis* changed less after the introduction of the mass vector control measures, and, as a result, the relative proportion of *An. arabiensis* increased markedly as the populations of *An. gambiae s.s.* and *An. funestus* collapsed, with *An. arabiensis* left as the predominant species after IRS (Fig. 4c).

## Discussion

Over the past 13 years (2007–2020), vector control interventions have been scaled-up substantially across Uganda. Whilst the impact of LLINs and IRS on epidemiological outcomes has been assessed routinely [4, 5, 7, 32, 53, 54], the effect of these interventions on malaria vector species is less commonly investigated. This study characterized vector species composition and density in three epidemiologically diverse settings from 2011 to 2016, while vector control interventions were implemented across the country by the Uganda Ministry of Health (National Malaria Control Division).

As expected, *Anopheles* densities were higher during the rainy season in all study sites, consistent with other studies [48, 55]. Prior to the widespread implementation of vector control interventions, *Anopheles* species were sympatric but composition varied between the sites, with *An. arabiensis* predominant in Walukuba (the lowest transmission site) and *An. gambiae* s.s*.* predominant in both Kihihi and Nagongera (the moderate and high transmission sites respectively). Delivery of LLINs was associated with significant declines in vector density for *An. funestus* in Walukuba, *An. gambiae s.s.* in Kihihi and in both *An. gambiae s.s.* and *An. arabiensis* in Nagongera. Addition of IRS to LLINs in Nagongera was associated with a decline in all vector species, albeit with a greater impact on *An. gambiae s.s.* and *An. funestus,* as reported elsewhere [56, 57]. Consequently, *An. arabiensis* became the predominant species in this area. Understanding the impact of vector control interventions on local malaria vector species is paramount for assessing gaps in current vector control tools.

Malaria vector control interventions, mainly LLINs and IRS have been associated with changes in sympatric *Anopheles species* composition in Uganda [10], and elsewhere in East Africa [11, 39, 43]. However, a shift in vector species composition and a decline in vector numbers has also been reported in absence of systematic vector control in north-east Tanzania [58, 59], which underscores the possibility of other causes for these changes, such as epidemics of mosquito pathogens, improvements in housing, and changes in climate and land use. Inherent differences in malaria vector ecological characteristics [25], host preference [17], and exophagic and exophilic behavior [29, 60, 61], could be a threat to vector control especially for *An. arabiensis* [41]. *Anopheles arabiensis* is considered to have a lower vectorial capacity than *An. gambiae s.s.* and *An. funestus* in parts of East Africa [38]. In other settings, however, where *An. arabiensis* is the principal vector, evidence of strong anthropophagic behaviour and outdoor malaria transmission have been described [60]. The opportunistic feeding behavior of *An. arabiensis*, enables this species to avoid contact with LLINs and walls sprayed with insecticides which are applied indoors [27, 60, 62, 63]. Empirical evidence shows that highly anthropophilic malaria vectors, such as *An. gambiae s.s.* and *An. funestus s.s.,* are more responsive to vector control, particularly IRS programmes [10, 39, 42]. A shift in biting patterns of *An. funestus*, however, including early morning biting [37, 64], and broad daytime biting [65], following introduction of LLINs has been documented.

Current vector control tools target highly anthropophagic and endophilic behaviour [63]. However, there is growing evidence of outdoor biting especially in *An. arabiensis* [62, 66], which poses a threat to vector control. A similar study, within the study area in Nagongera found a high proportion of *An. arabiensis* biting outdoors [10]. In this study, the combination of LLIN and IRS had a lower impact on *An. arabiensis* vector density compared to *An. gambiae s.s.* and *An. funestus*, making it the predominant malaria vector post-intervention. The impact of this apparent increase in *An. arabiensis* vector density on malaria transmission remains unclear, however. A similar study in Nagongera showed limited malaria transmission despite relatively abundant *An. arabiensis* [10]. In Kenya, there was a decline in malaria transmission following increased LLIN coverage, coincident with the replacement of primary malaria vectors, *An. gambiae s.s.* and *An. funestus* by *An. arabiensis* [39]. It is plausible that *An. arabiensis* may maintain residual transmission until the primary malaria vectors *An. gambiae s.s.* or *An. funestus* ‘bounce back’. This occurred in western Kenya, where previously dominant *An. funestus* was suppressed following long term use of LLINs, but then recovered, becoming the predominant vector again within a period of almost 20 years, possibly due to high levels of pyrethroid resistance in this species [67]. In a key example of vector control failure in Kwazulu Natal, previously ‘eliminated’ *An. funestus* was replaced by less endophilic *An. arabiensis*, but returned after almost 40 years, highly resistant to pyrethroids, and associated with a malaria resurgence in this area [68].

Outdoor biting behaviour of *An. arabiensis* poses a challenge to malaria vector control. Larval source management with microbial larvicides combined with LLINs has been shown to be protective against malaria infections in rural Kenya [69], and there are several measures including treating cattle with insecticide [60], use of odour-baited traps dispensing spatial repellents [70], and transfluthrin-treated chairs and ribbons [71], which could be deployed as control interventions in the future. In Uganda, there is still an information gap regarding the zoophilic behaviour of *An. arabiensis* and host choice in the presence of animals and humans. There is need for further research to assess the efficacy of interventions for controlling *An. arabiensis*.

This study had several limitations. First, the findings presented are from three sub-counties from only three districts. Thus, the study has limited geographical scope and the results may not be generalizable to other settings. Notably, however, the selected sites represented markedly different transmission settings, and all mosquito collections were made from randomly selected households after enumeration. Second, only indoor mosquito collections were done using light traps. Therefore, these results are subject to inherent biases presented by the mosquito trapping method used. Third, species-specific sporozoite data were not collected, therefore, implications to malaria control regarding residual transmission are implied. Within the study area, pyrethroid resistance was documented in both *An. gambiae s.s* and *An. arabiensis* [32], with evidence of carbamate resistance observed in *An. gambiae s.s.* from Nagongera and Kihihi [32]. However, the extent to which insecticide resistance affected mosquito survival under field conditions was not assessed. Study sites were not randomized to receive particular interventions; longitudinal measurements of mosquito density were made alongside vector control interventions delivered by the Uganda Ministry of Health. Whilst monthly rainfall measurements were used in the analysis and interpretation of the results, temperature and humidity data were unavailable for the study period.

*Anopheles* species composition may change from highly anthropophagic to less anthropophagic malaria vectors in response to vector control. However, the implications of these shifts in species composition on malaria transmission and control programmes are not well understood and require an in-depth examination of *Anopheles* species specific contribution to local malaria transmission. This study found that LLINs and IRS affected vector densities and species composition differently in different settings. Measuring absolute numbers of mosquitoes to quantify the impact of interventions instead of relying on relative proportions is important in order to understand the full picture.

## Conclusions

In areas of low- and moderate- malaria transmission large-scale deployment of LLINs resulted in substantial reductions in *An. gambiae s.s.* and *An. funestus s.l.* In the area of intense malaria transmission, the introduction of LLINs and IRS resulted in the near collapse of these main vectors, with *An. arabiensis* becoming the principal vector, but at lower densities than prior to wide-scale vector control. Measuring the impact of vector control interventions using absolute numbers of mosquitoes collected increased precision. These findings suggest that the impact of LLINs and IRS on the primary malaria vectors (*An. gambiae s.s.*, *An. arabiensis* and *An. funestus)* may be affected by the behaviour of these mosquito populations. Current vector control interventions are effective against malaria, but will not lead to elimination of the disease unless additional tools are included as supplementary interventions. Larval source management using chemical or microbial larvicides, combined with environmental management, could be used to improve control, especially in areas of high transmission.

## Data Availability

The data used are available from the corresponding author upon reasonable request. The PRISM1 dataset can also be accessed at https://clinepidb.org/ce/app/record/dataset/DS_0ad509829e.

## Abbreviations

LLIN: Long-lasting insecticidal nets
IRS: Indoor residual spraying
PCR: Polymerase chain reaction
EIR: Entomological inoculation rate
WHO: World Health Organization
MoH: Ministry of Health
